# Political orientation and COVID-19 policy preferences during the early pandemic: a comparative analysis of China and South Korea

**DOI:** 10.3389/fpubh.2025.1581798

**Published:** 2025-07-16

**Authors:** Zhou Fang

**Affiliations:** Graduate School of International Culture and Communication Studies, Waseda University, Tokyo, Japan

**Keywords:** COVID-19, China, South Korea, epidemic prevention policies, policy preferences, political tendencies

## Abstract

**Background:**

The COVID-19 pandemic posed an unprecedented challenge to governments and societies worldwide, highlighting the complex relationship between state policies and public acceptance during crises. This study examines how regime types, political tendencies, and social culture influence citizen’s preferences for epidemic prevention policies in China and South Korea.

**Materials and methods:**

A cross-sectional online survey was operated in May 2020 to citizens aged ≥ 20 years in China and South Korea. Using the stratified quota sampling method, This study collected 2,254 valid responses from China and 1,783 from South Korea. Moderated mediation effect analysis were conducted to explore the factors shaping public attitudes toward epidemic control measures.

**Results:**

There are clear differences in policy preferences between the two countries. Chinese citizens were more likely to support strict, government-led measures, while South Korean respondents showed lower levels of support. Political tendency was the strongest predictor of policy preferences in both countries, with those favoring greater government authority more likely to support strict epidemic control measures. While government satisfaction significantly influenced policy acceptance in China, its impact was minimal in South Korea. Additionally, collectivism moderated the relationship between political tendency and policy preferences. In highly collectivist environments, social norms reinforced support for control measures, while in more individualist contexts, personal political beliefs had a stronger influence on policy acceptance.

**Conclusion:**

This study provides important evidence that citizen preferences for epidemic prevention are shaped by complex interaction between political institutions, cultural values, and individual beliefs. These findings can inform more effective communication strategies and policy design when governments prepare for future pandemic.

## 1 Introduction

The COVID-19 pandemic emerging in late 2019 spread rapidly to more than 200 countries around the world, posing a great challenge to global public health (1–3). Governments have implemented various Non-Pharmaceutical Interventions (NPIs) to control the diffusion of the pandemic. For example, the Chinese government adopted a series of strict prevention and control strategies to suppress the transmission of the virus (4). By doing mass nucleic acid testing, health code management, and entry quarantines, the Chinese government successfully decreased infection and mortality rates after the first outbreak in Wuhan. However, the high transmissibility caused by SARS-CoV-2 variants led China to shift to the “dynamic zero-COVID” policy. Unlike the early lockdown measures, this strategy imposed stricter controls and regulations, reinforced through technological upgrades and mobilization of multiple bureaucrat units to track, monitor and restrict people’s movement (5). These policies came at an expensive cost. After nearly a year of outbreaks caused by the highly contagious Omicron variant, China ultimately lifted these restrictions in late 2022.

South Korea was one of the first countries to successfully control the pandemic (6, 7). Instead of imposing aggressive lockdowns or complete travel bans, the South Korean government focused on expanded diagnostic testing, rigorous contact tracing, and identifying exposed individuals (8). These measures effectively controlled the first wave of the pandemic. However, the relaxed prevention policies led to multiple subsequent outbreaks. The South Korean government finally shifted to a *living with the COVID-19* policy in early 2022, allowing the country to exit the epidemic control phase earlier than China.

The China and South Korea cases show the complexity of epidemic prevention and control, and how to make prevention policy more effectively has received much attention. Research has found that public acceptance of epidemic prevention policies varies across countries (9). Chinese citizens generally supported government-led measures, meanwhile South Korea also showed good compliance with government recommended policies such as social distancing, wearing masks (10, 11).

Despite extensive research on pandemic response strategies, there remains a critical gap in understanding how citizens in different political systems perceive and accept various epidemic prevention policies. While existing studies have documented policy effectiveness from epidemiological perspectives, limited comparative research examines public acceptance of stringent measures across different governance models. This study addresses this gap by comparing citizen preferences for epidemic prevention policies between China’s centralized governance system and South Korea’s pluralism part governance structure.

The effectiveness of response policies was influenced not only by the nature of the epidemic and prevention strategies but also by factors such as government systems, social culture, and public trust. Therefore, studying Chinese and South Korean citizens’ preferences for anti-epidemic policies and the factors influencing them has both academic value and practical significance. Understanding how public health governance models operate under different political systems can provide insights for future public health crisis management.

Against the backdrop, this study asks two research questions:

(1) How does citizens’ acceptance of epidemic prevention policies differ between China and South Korea?
(2) Through what pathways do regime type, government satisfaction, cultural and risk perception shape citizens’s support for containment measures?

To respond to the above questions, this study examines the differences in citizens’ preferences for epidemic prevention and control policies between China and South Korea. The remainder of this paper is organized as follows. First, it summarizes epidemic prevention and control policies in China and Korea and examines the effects of government systems, government trust, and socio-cultural factors on policy acceptance. Next, it describes the questionnaire design, sample selection, variable measurements, and data analysis methods. Then, it statistically examines policy preference differences and their influencing factors. Finally, it summarizes key findings, provides policy recommendations, discusses research limitations, and suggests directions for future research.

The practical significance of this study is manifested in three aspects. First, it provides cross-national evidence to understand public compliance behavior during the pandemic, complementing existing public health governance research. Second, it helps explain why global pandemic response strategies vary widely, highlighting the importance of designing political and cultural interventions. Third, it offers insights for countries to formulate more scientific, and efficient pandemic response policies, seeking an optimal solution between effective disease control and citizens’ support.

## 2 Literature review

This section will review the literature related to this study and focus on the following themes: (1) regime type and anti-epidemic policies; (2) government trust and policy acceptance; (3) socio-cultural influences on policy preferences; and (4) the shaping of citizens’s attitudes by risk perception and the political tendency. By sorting out these key concepts, this study will develop a theoretical framework to explain the differences between Chinese and Korean citizens’ preferences for the new crown epidemic prevention policy.

### 2.1 Epidemic prevention and control policy shifts in China and Korea

The global response to the COVID-19 epidemic has gone through several stages, with countries continuously adjusting their prevention policies as the virus mutated and understanding of the disease deepened. These epidemic control strategies can be divided into two types: one type is a containment strategy, and the other is a mitigation strategy (8). A containment strategy focuses on disease prevention and the control of infectious diseases from three aspects: infectious sources, transmission routes, and susceptible populations (5, 12, 42). It aimed to break the chain of transmission through a combination of aggressive test-and-isolate policy (identify and isolate all infectious persons, including those with mild illness) The confirmed cases and suspected cases were treated intensively until the medical observation period was complete.

Whereas, a mitigation strategy focuses on reducing the transmission rate, asserting that the spread of COVID-19 cannot be completely interrupted and can only be slowed when the population forms an adequate immune barrier and the intensity of the epidemic decreases to become a seasonal infection, such as influenza. It aimed to reduce death tolls by focusing on the medical care of severe cases while relying on social distancing to flatten the curve of epidemic impact on healthcare systems. Moreover, the mitigation strategy prioritizes hospitalization for severe cases or those with the underlying disease rather than early detection of all cases, isolates and treats mild cases, or sereness and manages close contacts.

China and Korea have modified their strategies over time in response to the changing situation. The details of their policies are presented in Tables 1, 2 (13, 14).

**Table 1:** Chinese prevention and control policies.

Period	Time	Main strategies
Wuhan outbreak	2020.1 ~ 2020.3	Lockdown, nucleic acid tests, health code
Stable in domestic	2020.4–2021.6	Immigration quarantine, targeted blockade, vaccinations
Delta wave	2021.7–2021.12	Partial blockade, dynamic zero policy
Omicron wave	2022.1–2022.12	Strict blockade, large-scale nucleic acid testing
Post-epidemic	2022.12–Now	Cancelation of the zeroing policy

**Table 2:** Korean preventive and control policies.

Period	Time	Main strategies
Early period	2020.1–2020.4	Testing, tracing, treatment (3 T strategies)
Repeated outbreaks	2020.5–2021.6	Four stages of social distancing measures
Delta wave	2021.7–2021.12	Population immunity, with COVID-19 strategies
Omicron wave	2022.1–2022.11	Relaxation of epidemic prevention policies
Post-epidemic	2022.11–Now	Remove all restrictions

Tables 1, 2 reveal that both China and South Korea adopted a rapid response and strict control strategy to contain the epidemic in its early stages. China took measures such as large-scale lockdown and mandatory isolation to quickly curb the spread of the virus. In contrast, South Korea did not implement city-scale lockdowns, it adopted large-scale nucleic acid testing and patient tracking.

Between May 2020 and June 2021, China succeeded in keeping the domestic outbreak at a low level through a strict entry quarantine policy and achieved a rapid economic recovery. Meanwhile, South Korea adopted a more relaxed approach, implementing nucleic acid testing for cross border enters and home quarantine measures. Economic activity partially recovered but was affected by a recurring outbreak (32–34).

In the second half of 2021, the spread of the delta variant led to a new outbreak worldwide. China Implemented a *dynamic zero* policy, further strengthening containment measures and accelerating vaccination. Although the policy was effective in controlling the outbreak, it had a significant impact on economic and social life. Business operations and residents’ working lives were largely disrupted. During this period, Korea attempted to transition to a *living with the virus* approach by relaxing social distancing measures and mask wearing order. However, due to a resurgence of cases caused by the Delta variant, the South Korean government temporarily reinstated social distancing measures between July and December 2021.

In 2022, the emergence of the Omicron variant led to renewed lockdowns in several Chinese cities. China continued to adhere to its *dynamic zero-COVID* policy, enforcing strict containment measures and large-scale testing. Due to growing economic and social pressures, the policy was adjusted in December 2022. South Korea also faced a huge challenge from Omicron, but due to the low fatality rate of this variant, the South Korean government adjusted its policy in early 2022, removing vaccine passes and outdoor mask orders, entering the reopen phase earlier than China.

From a global perspective, both China and South Korea’s early pandemic response could be characterized as variants of stringent containment strategies, particularly when compared to the more relaxed approaches initially adopted in many Western countries (35, 39). However, important distinctions existed between the two approaches. China’s response emphasized mandatory, centralized control with extensive use of lockdowns and movement restrictions (4), while South Korea relied more heavily on technology-enabled contact tracing, voluntary compliance, and targeted interventions without implementing city-wide lockdowns (15). These differences, while subtle from a global standpoint, reflected great different governance ideology that deserve closer examination.

### 2.2 Regime type and the preference for epidemic prevention policies

Since the outbreak of COVID-19, extensive research has been conducted on the factors influencing the effectiveness of epidemic prevention policies. In addition to studies on virus characteristics and healthcare resources, many scholars have examined the topic from the perspective of government governance, social culture, economic factors, and policy acceptance (6, 10, 16, 17).

The role of government systems in epidemic prevention has attracted significant attention. Studies show that different political systems exhibit distinct approaches to policy formulation and implementation (8). China’s one-party system is characterized by centralized decision-making and unified policy implementation with authoritarian features. Countries like China tend to enforce large-scale, mandatory containment measures such as lockdowns, compulsory quarantines, and mass nucleic acid testing, allowing for efficient policy execution (18–20, 42). In contrast, democratic regimes generally require more time before making policy decisions and face more constraints during implementation (7, 12, 15). For example, South Korea’s multi-party democracy features a distributed decision making process, and its 4 year presidential election cycle may reduce administrative efficiency. As a result, they often adopt more flexible, individual volunteering measures, such as social distancing recommendations, voluntary testing, and encouragement of vaccination.

Chung et al. (10) found that citizens under authoritarian regimes may prioritize security over liberty, whereas citizens in democratic settings tend to be more skeptical of stringent government interventions. This institutional contrast not only manifests in the methods and intensity of policy enforcement but also affects levels of public trust in government and interpretations of individual freedom.

Research on the impact of government systems on epidemic control suggests that authoritarian regimes can suppress virus transmission more quickly in the early stages of an outbreak. However, their containment measures tend to have a greater long-term impact on the economy and society (5). Democratic countries, while facing initial challenges in managing the outbreak, demonstrate greater flexibility in adjusting policies over time, leveraging public autonomy and adaptive strategies.

### 2.3 Government satisfaction and policy preferences

Government satisfaction emerges as critical factors in the relationship between governance systems and policy preferences. Studies have shown that higher trust in the government increases citizens’ willingness to cooperate with epidemic prevention measures. However, government trust levels have varied across countries during the pandemic.

Zhu (4) study found that overall Chinese citizens trust the government’s epidemic prevention policies and are willing to comply with health management measures. In contrast, South Korean citizens have lower trust in their government. Li and Hong (21) longitudinal study found that Chinese citizen’s confidence in the political system was significantly linked to their attitudes toward the *dynamic zero-COVID policy.* They founded that citizens with higher political confidence were more supportive of strict containment measures. Research by Kang et al. (6) revealed that in the early stages of the pandemic, the South Korean government gained some public trust due to its effective testing and contact tracing system. However, in early 2022, the *living with the virus* policy led to repeated outbreaks, causing some South Koreans to believe the government had failed to protect high-risk groups, which resulted in declining trust levels.

Comparative studies on government trust in China and South Korea suggest that citizens with higher trust in the government are more likely to support strict containment measures, whereas those in countries with lower trust levels are more likely to oppose such policies (22). Additionally, the transparency of epidemic policies influences public trust (38). Vu (23) found that government disclosure of pandemic data and infection tracking enhances public trust in government measures. Research also indicates that government trust acts as a mediating factor between government systems and policy acceptance—citizens with higher trust levels are more likely to accept government-imposed epidemic prevention policies (10).

### 2.4 Cultural and risk perception on policy preferences

**C**ultural factors have also played a crucial role between government trust and policy preferences through value-based filtering of information and differential weighting of policy evaluation criteria. Research on cultural dimensions reveals that collectivist cultures tend to emphasize compliance with government policies, whereas individualist cultures show preferences for policies and prioritize personal rights (24, 37). Studies examining Confucian cultural influences reveal distinct patterns in societies sharing this cultural heritage, including enhanced respect for hierarchical authority, emphasis on collective responsibility, and preference for harmonious social relationships (40). These cultural characteristics create specific moderation patterns where government trust more strongly predicts policy acceptance compared to societies with different cultural foundations. Both China and South Korea belong to East Asian societies often characterized by strong collectivist values, which may encourage citizens to cooperate with or support governmental actions during major public crises (41).

Risk perception refers to an individual’s subjective identification of risk that they may face, shaped by uncertainty and fear (25). Research by Liao et al. (26) found that a key characteristic of pandemic viral infections is the functional fear they can instill across vast segments of the population. Risk perception is a negative emotional response, often leading to extreme emotive avoidance toward specific stimuli. It has been linked to clinical phobias and social anxiety disorders, indicating that widespread public fear caused by pandemic can result in significant mental distress at the population level (27). Additionally, Studies have shown that a higher perceived personal risk of infection correlates with an increased likelihood of engaging in preventive behaviors, such as handwashing and social distancing (28).

The previous literature demonstrates that governance systems, government satisfaction, cultural values, and risk perception are all critical factors that significantly influence citizens’ attitudes toward public health policies during crisis periods. However, current studies have rarely examined how citizens in different countries accept various levels of epidemic prevention policies. Furthermore, the mechanisms through which social culture and pandemic perception influence policy acceptance remain insufficiently explored.

## 3 Methodology

**Figure 1:**
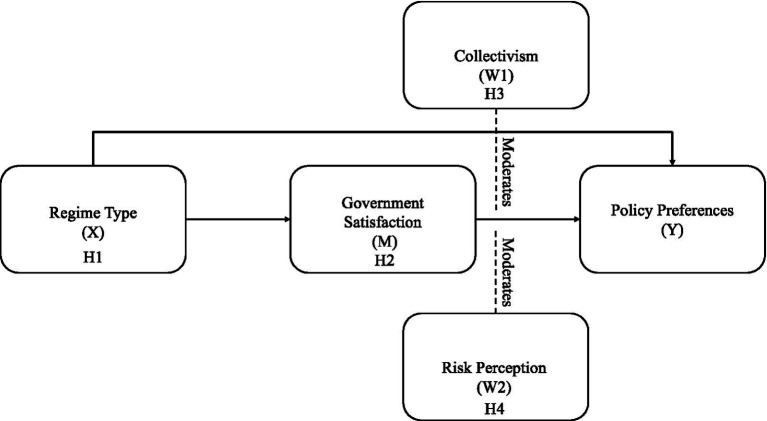
Policy preferences mediation model.

Building on the literature reviewed above, this study constructed a moderated mediation model to link regime type to citizen’s support for COVID-19 preventative measures through government satisfaction and test two socio-psychological contingencies-collectivist orientation and risk perception. In this model, Regime type served as the independent variable (X), *Political satisfaction* as the mediating variable (M) and *Policy preference* as the dependent variable (Y). *Collectivism* and *Risk perception* were included as moderators (W1; W2). Figure 1 presents the expected paths, and the corresponding hypotheses are stated below.

*H1*: Chinese citizens are more inclined to support government-led mandatory epidemic prevention policies than South Korean citizens.

*H2*: Government Satisfaction mediates the relationship between political systems and policy preferences—higher government trust increases citizens’ support for government-led epidemic prevention policies.

*H3*: Social culture moderates the impact of government trust on policy preferences—under more collectivist cultures, government trust has a stronger influence on policy support.

*H4*: Risk perception moderates the relationship between government trust and policy preference. In authoritarian regimes, lower fear of the pandemic is linked to higher support for government policies.

### 3.1 Participants

This study conducted a cross-sectional comparative survey design to examine citizen preferences for epidemic prevention policies in China and South Korea. Online surveys was conducted in the two countries using online platforms (China: Wenjuxin; Korea: Google Forms) in May 2020. Adult citizens living in the domestic region aged 20 and above were collected. A total of 4,037 valid responses were collected: 2,254 valid responses were collected from China and 1,783 from South Korea.

Table 3 presents the demographic information of respondents. In the Chinese sample, 51.4% of the participants were female, where females constituted 51.1% in Korea. Participants in both countries were generally well-educated; in China, over 85% had gained a college degree or higher, while in South Korea, approximately 81.7% had achieved the same level of education.

**Table 3:** Demographic characteristics of participants.

Demographic categories	Type	China (*N* = 2,250)		South Korea (*N* = 1783)	
		*N*	Percentage (%)	*N*	Percentage (%)
Gender	Male	1,093	48.6	871	48.9
	Female	1,157	51.4	912	51.1
Age	20–29	655	29.1	447	25.1
	30–39	832	37	396	22.2
	40–49	377	16.8	402	22.5
	50+	386	17.2	538	30.2
Education	Junior high school graduate	32	1.4	32	1.8
	High school graduate	303	13.5	294	16.5
	College graduate	1740	77.3	1,264	70.9
	Graduate school	175	7.8	193	10.8
Salary	Low income	394	17.5	542	30.4
	Middle income	601	26.7	642	36.0
	Upper middle income	656	29.2	453	25.4
	High income	599	26.6	146	8.2

### 3.2 Instruments

A custom-designed questionnaire for this study was meticulously crafted. The questionnaire has 2 sections with a total of 29 items. Section A gathered demographic details, Section B captured the participants’ perception using a 4-point Likert scale (ranging from 1 “Strongly Disagree/Oppose” to 4 “Strongly Agree/support”). The questionnaire can be seen in Supplementary material.

*Policy preferences* were assessed using 12 items (Q7-Q18). Items were categorized into two categories: *Prohibit* (Q7–Q11) (e.g., “Prohibit all non-essential movement of people outside of their homes and ban all large gathering”) and *Control* (Q11–Q18) (e.g., “Detain any individual exhibiting coronavirus-like symptoms and quarantine them in a government facility for at least 2 weeks”). Composite scores were calculated as the mean of relevant items, with higher scores indicating greater policy support. Scores ranged from 1.0 to 4.0 for each policy category.

*Government satisfaction* as the mediating variable, was assessed using 2 items (Q24–Q25, e.g., “Do you approve or disapprove of government’s overall job performance thus far”). C*ollectivism* was assessed using Hofstede’s Value Survey Module, which included Four items (Q1–Q3), such as “How well does the term “Chinese/Korean” describe you?” The second moderating variable, *Risk Perception*, was measured using two questions (Q4-Q5), including “I am afraid that I will get the coronavirus.” And “I am afraid that people I care about will get the coronavirus.” To access personal and interpersonal COVID-19 concerns.

Although the two countries have different political systems, this does not mean that citizens fully agree with all the principles promoted by their respective governments. To measure the political tendency among citizens in both countries, this study designed five questions (Q19–Q23) based on the political spectrum (29) to evaluate individuals’ willingness (e.g., “fighting the coronavirus is more important than upholding the law”). Higher scores indicate great willingness to prioritize epidemic prevention over law and economic development and are more willing to grant greater authority to the government to control the pandemic.

The reliability of all variables was confirmed with a high Cronbach’s alpha value of 0.854. Detailed reliabilities coefficients for each variables are listed in Table 4. Demographic variables included gender (female = 0, female = 1), age (1 = 20–29, 2 = 30–39. 3 = 40–49. 4 = 50–59, 5 = 60 or above), education level (1 = middle or below, 2 = high school, 3 = bachelor’s degree, 4 = graduate degree), and household income (1 = low income, 2 = middle income, 3 = middle high income, 4 = high income).

**Table 4:** Reliability statistics.

Variables	Cronbach’s alpha		No. of items
	China	Korea	
Policy preferences	0.843	0.829	12
Government satisfaction	0.759	0.906	2
Collectivism	0.647	0.612	4
Risk perception	0.853	0.896	2
Political tendency	0.704	0.806	5
Sum	0.854	0.854	25

### 3.3 Data processing

SPSS 29.0.2.0 was used to conduct descriptive, correlation and regression analyses. PROCESS was used for moderated mediation analyses. In the path analyses, all variables were standardized and 5,000 resamples were used to obtain confidence intervals (CIs) by bootstrapping.

## 4 Results

### 4.1 Descriptive statistics and correlations between variables

Table 5 presents the descriptive statistics for each variable. Chinese respondents reported significantly higher levels of government satisfaction (*M* = 3.67, *SD* = 0.51), collectivist (*M* = 3.61, *SD* = 0.43), and political tendency (*M* = 3.13, *SD* = 0.45) compared to Korean respondents (government satisfaction: *M* = 2.94, *SD* = 0.87; collectivism: *M* = 3.27, *SD* = 0.47; political tendency: *M* = 2.83, *SD* = 0.61). Chinese respondents also scored higher on both policy preference variables—Prohibit (*M* = 3.37, *SD* = 0.45) and Control (*M* = 3.45, *SD* = 0.46)—than their South Korean counterparts (Prohibit: *M* = 3.04, *SD* = 0.50; Control: *M* = 2.95, *SD* = 0.53). There was no significant difference in risk perception between the two countries, suggesting that it may not have been the primary factor influencing differences in policy preferences during the early stages of the pandemic.

**Table 5:** Result of respondents’ attitudes toward policies.

Variables	China	Korea	*Sig*	*t*
	M (SD)	M (SD)		
Satisfaction	3.67 (0.51)	2.94 (0.87)	<0.001	−33.06
Collectivism	3.61 (0.43)	3.27 (0.47)	<0.001	−23.42
Risk Perception	2.84 (0.80)	2.80 (0.73)	0.001	−1.53
Political tendency	3.13 (0.53)	2.83 (0.61)	<0.001	−16.39
Prohibit	3.37 (0.45)	3.04 (0.50)	<0.001	−21.63
Control	3.45 (0.46)	2.95 (0.53)	<0.001	−31.91

Table 6 presents the correlation results of both China and Korea. The results indicate both similarities and significant cultural and institutional differences in the factors influencing policy preferences in the two countries.

**Table 6:** Correlations between policy preferences and variables.

Variables	Prohibit		Control	
	China	Korea	China	Korea
Satisfaction	*r* = 0.311, *p* < 0.001	*r* = 0.068, *p* < 0.005	*r* = 0.440, *p* < 0.001	*r* = 0.243, *p* < 0.001
Collectivism	*r* = 0.335, *p* < 0.001	*r* = 0.180, *p* < 0.001	*r* = 0.432, *p* < 0.001	*r* = 0.209, *p* < 0.001
Risk perception	*r* = 0.026, *p* = 0.218	*r* = 0.189, *p* < 0.001	*r* = 0.002, *p* = 0.942	*r* = 0.132, *p* < 0.001
Political tendency	*r* = 0.467, *p* < 0.001	*r* = 0.459, *p* < 0.001	*r* = 0.450, *p* < 0.001	*r* = 0.557, *p* < 0.001

In the Chinese respondents, government satisfaction was positively correlated with both prohibitive and control-oriented epidemic prevention policies (*r* = 0.311, *p* < 0.001; *r* = 0.440, *p* < 0.001), indicating that citizens with higher government satisfaction were more likely to support strict prevention measures. Similarly, collectivism showed a significant positive correlation with both policy preferences (*r* = 0.335, *p* < 0.001; *r* = 0.432, *p* < 0.001), suggesting that individuals with stronger collectivist values were more accepting of strict prevention measures. The strongest correlation was in the political tendency factor (*r* = 0.467, *p* < 0.001 *r* = 0.450, *p* < 0.001), indicating that those who support greater government authority were more likely to endorse strict epidemic control measures. Notably, risk perception was not significantly correlated with policy preference in the Chinese sample (*p* > 0.05).

In the South Korean sample, the correlation between government trust and policy preference was weaker compared to China (Prohibit: *r* = 0.068, *p* < 0.005; Control: *r* = 0.243, *p* < 0.001). By contrast, collectivism remained positively correlated with policy preference (*r* = 0.180, *p* < 0.001; *r* = 0.209, *p* < 0.001), though its influence was weaker than in China. Additionally, *political tendency* remained a strong predictor of policy preference in South Korea (*r* = 459, *p* < 0.001; *r* = 0.557, *p* < 0.001), reinforcing the idea that political stance was a key factor in determining support for government epidemic policies.

To examine the factors associated with public support for COVID-19 prevention policies, a multiple linear regression model was constructed using a combined dependent variable: p*olicy preference* which is derived from the *Prohibit* and *Control* items. Table 7 represents the regression results for China and South Korea.

**Table 7:** Results of multiple linear regression analysis on factors associated COVID-19 policy.

Variables	China		South Korea	
	Coefficient	SE	Coefficient	SE
Satisfaction	0.154***	0.017	0.004	0.011
Collectivism	0.227***	0.020	0.105***	0.020
Risk perception	−0.005	0.009	0.054***	0.013
Political tendency	0.314***	0.014	0.404***	0.016
Gender	−0.004	0.014	0.006	0.020
Age	0.009	0.007	−0.009	0.009
Education	−0.005	0.015	−0.044*	0.016
Income	−0.007	0.007	−0.014	0.011

China *N* = 2,250, *R*^2^ = 0.366; Korea *N* = 1783, *R*^2^ = 0.332. ***p < 0.001, **p < 0.05, *p < 0.1.

Across both countries, political tendency emerged as the strongest and most consistent predictor of policy preferences (China: *β* = 0.314, *p* < 0.001; South Korea: *β* = 0.404, *p* < 0.001), indicating that individuals who believe the government should wield more authority are significantly more likely to support strict epidemic prevention measures. Collectivism also had a positive and statistically significant effect on policy preference in both countries (China: *β* = 0.227, *p* < 0.001; South Korea: *β* = 0.105, *p* < 0.001), suggesting that collectivist values play a meaningful role in shaping policy attitudes, regardless of regime type.

Interestingly, government satisfaction had a significant positive association with policy preference in China (*β* = 0.154, *p* < 0.001), but this relationship was not significant in South Korea (*β* = 0.004, *ns*). This contrast implies that in central governance contexts, public trust in the governments may be a more critical determinant of policy compliance, while in democratic contexts, its influence may be diluted by other institutional or individual factors. Risk perception was only significantly related to policy preference in South Korea (*β* = 0.054, *p* < 0.001), whereas it was not a significant predictor in China (*β* = −0.005, *ns*). This may be due to the early stage of the pandemic during data collection, when public understanding of COVID-19 in China and South Korea were limited.

Demographic variables, gender, age, education, and income exhibited no consistent or substantial effects across either country. Only education had a modest negative association with policy preference in South Korea (*β* = −0.044, *p* < 0.1), while none of the demographic factors were significant predictors in China. This suggests that political ideology and cultural orientation played a more prominent role than socioeconomic status in shaping early pandemic policy attitudes.

### 4.2 Moderated mediation effect analysis

**Figure 2:**
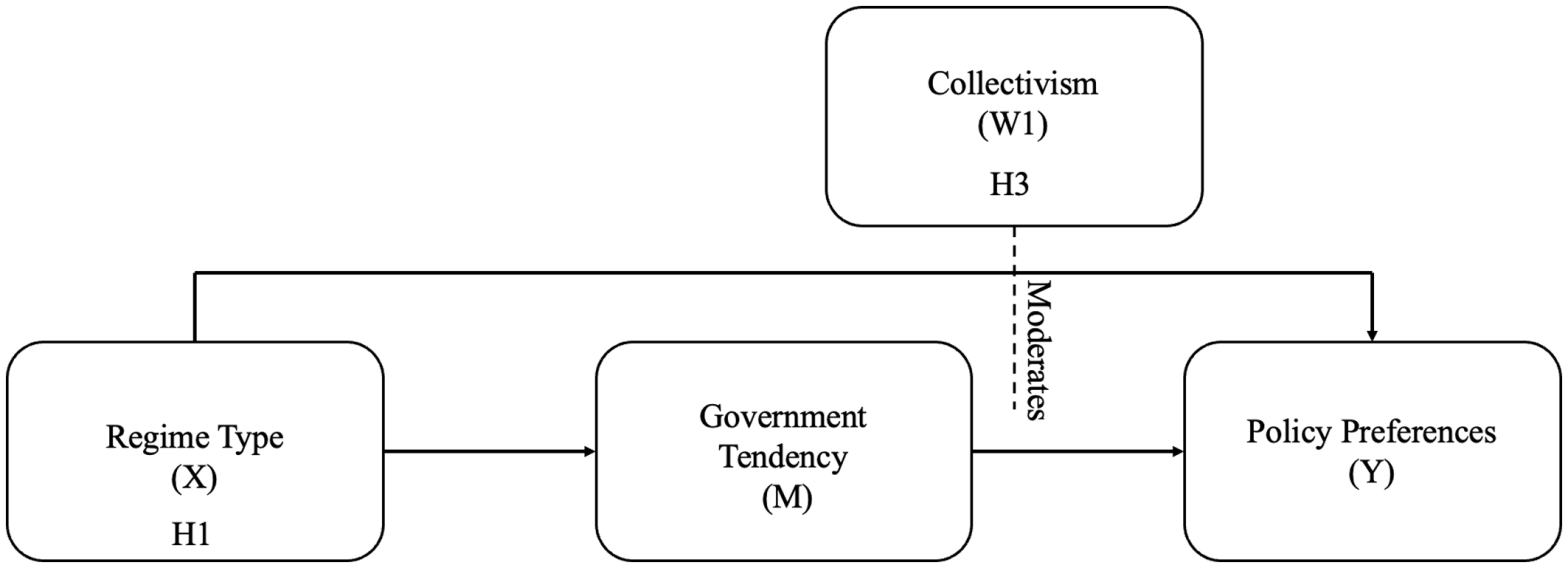
Refined policy preferences mediation model.

Based on the previous data analysis results above, we refined the model by changing the *political tendency* as the mediating variable (M) and removing the risk perception as the moderating variable (W2). The refined model is illustrated in Figure 2.

The direct effect of regime type on policy preference was positive and statistically significant (*β* = 0.2329, *p* < 0.001), indicating that Chinese citizens are more likely than South Korean citizens to support government-led epidemic prevention policies. *Political tendency* had a strong and significant positive association with policy preference (*β* = 0.5088, *p* < 0.001), confirming that individuals who support stronger governmental authority are more likely to endorse stricter epidemic control measures. *Collectivism* also showed a significant positive impact on policy preference (*β* = 0.3181, *p* < 0.001), indicating that individuals with stronger collectivist values are more likely to accept stricter government-led pandemic measures.

Importantly, the interaction term (*Political tendency × Collectivism*) was statistically significant and negative (*β* = −0.0381, *p* < 0.05), indicating a moderating effect. Specifically, the positive association between political tendency and policy preference is weaker among individuals within higher collectivist society. In contrast, among individuals with lower collectivist orientation, political ideology exerts a stronger influence on their policy preferences. The moderated mediation effect analysis detail results are presented in Table 8.

**Table 8:** Moderated mediation effect result.

Variable	Coefficient	SE	*t*	*Sig*	LLCI, ULCI
Country	0.2329	0.0126	18.4881	< 0.001	[0.2082, 0.2576]
Political tendency	0.5088	0.0674	7.5529	< 0.001	[0.3767, 0.6409]
Collectivism	0.3181	0.0567	5.6118	< 0.001	[0.2070, 0.4293]
Ideology × collectivism	−0.0381	0.0192	−1.9802	< 0.05	[−0.0759, −0.0004]

Overall, *political tendency* is the most influential factor in explaining support for epidemic prevention policies. In both China and South Korea, citizens who support greater government authority are more likely to accept government-led epidemic prevention policies. However, collectivism moderates this relationship in different societal contexts. In low-collectivism societies, political tendency has a stronger impact on policy preference, meaning that individuals rely more on their political beliefs to assess the legitimacy of government policies. In high-collectivism societies, the influence of political tendency is weaker, as citizens are more likely to follow social norms and accept government-led measures. This supports the H2 and H3 that political and cultural variables interact in complex ways to shape public attitudes toward crisis governance.

## 5 Discussion

The COVID-19 pandemic posed great challenges for societies and governments worldwide, revealing critical dynamics between state capacity, political ideology, and cultural norms in shaping public compliance. Drawing on survey data from China and South Korea collected during the early pandemic phase (May 2020), this study contributes to our understanding of how different governance models can effectively manage public health crises. While research has shown that both authoritarian and democratic systems face distinct challenges in pandemic response.

H1 was strongly supported, confirming that Chinese citizens demonstrated significantly higher support for both prohibitive and control-oriented epidemic prevention policies (Prohibit: *M* = 3.37; Control: *M* = 3.46) compared to their South Korean counterparts (Prohibit: *M* = 3.04; Control: *M* = 2.96). This findings aligns with previous literature emphasizing centralized governance and societal stability in China as key facilitators of mass policy compliance (19, 30, 36).

H2 received partial support. Government satisfaction showed differential effects: it was a significant predictor in China (*β* = 0.154, *p* < 0.001) but not in South Korea (*β* = 0.004.*ns*). These results indicate that in centralized governance systems, trust in government institutions may be more directly linked to policy compliance, while in democratic contexts, other factors such as individual political ideology may play more important roles. South Korea’s democratic context may have diluted the influence of trust. Democratic pluralism often institutionalized opposition, complicating the relationship between satisfaction and compliance. As noted in previous literature, low governance satisfaction in democracies may reflect partisan opposition rather than resistance to specific policies. The politicization of health measures in competitive systems may explain the observed weaker effect.

One of the most important findings in this study is that political tendency emerged as the strongest predictor of policy preferences across both political systems (China: *β* = 0.314; South Korea: *β* = 0.404). This suggests that citizen’s fundamental beliefs about the appropriate government authority transcend specific institutional arrangements or cultural contexts. This finding has important implications for understanding how public perception of crisis policies are shaped not merely by institutional factors, but by deeper ideological orientations about state power and individual rights.

H3 was supported, the result showed that collectivism moderated the relationship between political attitudes and policy preferences. The negative interaction effect (*β* = −0.0381, *p* < 0.05) indicates that in more collectivist contexts, political ideology has a weaker direct impact on policy preferences, while in less collectivist societies, individual political beliefs more strongly determine policy support.

This finding aligns with research on cultural influences in health communication, which shows that collectivist societies tend to emphasize group harmony and compliance with social norms during crisis periods (40). In highly collectivist societies like China and South Korea (China, *M* = 3.61; South Korea: *M* = 3.27), citizens may be more likely to support government measures based on social expectations and group solidarity, regardless of their individual political leaning. The sustained significance of collectivism as a direct predictor in both countries (China: *β* = 0.227; South Korea: *β* = 0.105) reinforces the importance of cultural values in shaping crisis responses preferences.

Although both China and South Korea share an East Asian cultural tradition, changes in social structures and political institutions have shaped South Korea into a society where individualism and collectivism coexist (10). The analysis result indicated that Korean respondent’ policy preferences scores lower than their Chinese counterparts. This finding contributed to the growing literature on how cultural factors influence public acceptance of government interventions during pandemics (7, 10, 31, 40).

H4 was not supported, as risk perception showed limited and inconsistent effects across the two countries. Risk perception was only significant in South Korea (*β* = −0.0381, *p* < 0.001) and had no significant relationship with policy preferences in China. This unexpected finding may reflect the timing of data collection. During the early stages of the pandemic, public understanding of COVID-19 risks was still developing and risk perception may not have fully crystallized.

These findings have several implications for crisis governance and public health policy strategies. First, the primacy of political tendency suggests that effective crisis management must account for citizens’ fundamental beliefs about government authority and individual rights. Second, the differential role of government satisfaction between political systems indicates that trust-building strategies may need to be made to specific governance contexts. In centralized systems, maintaining a high level of institutional trust may be important for policy compliance, while in democratic contexts, building support may require greater emphasis on transparency and accommodation of diverse viewpoints. Third, the moderating effect of collectivism suggests that cultural sensitivity in policy design is essential. In order to control new pandemics in the future, public health policies that align with cultural values regarding collective responsibility may achieve greater acceptance than those that emphasize individual choice.

The study has some limitations. The data were collected during the early stages of the pandemic, when understanding of the virus and its impacts was still developing. Later research has shown that public attitudes toward pandemic policies evolved significantly as the crisis progressed and the pandemic existed for a long time (30). The study focused on only two East Asian countries limits broader generalizability. Regarding cultural attribution, we cannot definitively conclude that observed differences result from cultural rather than other factors such as economic or historical factors. However, several features strengthen our cultural interpretation. We measured collectivism at the individual level rather than assuming country-level differences, and the moderation analysis shows that collectivism affects attitude-behavior relationships within countries. Future research should explore how policy preferences changed over time, particularly during later waves of the pandemic. Comparative work includes countries with more diverse political and cultural backgrounds would provide deeper insights into the relationship between crisis governance and policy preferences.

## 6 Conclusion

Through cross-national data analysis, this study reveals how government systems, political orientation, social culture, and individual characteristics collectively influence citizens’ preferences for epidemic prevention policies. The findings indicate that different political systems may require different approaches to building public support for health measures. The primacy of political ideology in shaping policy preferences, combined with the moderating effects of cultural values, highlights the importance of understanding citizen attitudes and beliefs in public health design. As governments prepare for future pandemics, these insights can inform more effective communication strategies and policy frameworks.

## Data Availability

The raw data supporting the conclusions of this article will be made available by the authors, without undue reservation.
