# Supplementary material for: Political orientation and COVID-19 policy preferences during the early pandemic: a comparative analysis of China and South Korea
Source: Front Public Health. 2025 Jul 16;13:1581798. doi: 10.3389/fpubh.2025.1581798 (PMC12307314; doi:10.3389/fpubh.2025.1581798)
Supplement: Supplementary file 1 [file Table_1.docx]

covid19 questionnaires

1. What is your gender?

- Male
- Female

1. How old are you, generally?

- 20-29
- 30-39
- 40-49
- 50-59
- 60+

1. What best describes your highest level of education?

- Middle school or below
- High school
- Four-year college graduate
- Graduate school graduate

4. What is your total combined *household* income before taxes? Like the rest of the survey, this question is completely confidential and will be used only to classify the survey responses. (China)

- Less than RMB 30,000
- RMB 30,000 or more, but less than RMB 50,000
- RMB 50,000 or more, but less than RMB 70,000
- RMB 70,000 or more, but less than RMB 90,000
- RMB 90,000 or more, but less than RMB 110,000
- RMB 110,000 or more, but less than RMB 130,000
- RMB 130,000 or more, but less than RMB 150,000
- RMB 150,000 or more, but less than RMB 170,000
- RMB 170,000 or more

1. What is your total combined *household* income before taxes? Like the rest of the survey, this question is completely confidential and will be used only to classify the survey responses. (South Korea)

- ¥0 （no income）
- Less than ¥1 million
- ¥1 million or more, but less than ¥2 million
- ¥2 million or more, but less than ¥3 million
- ¥3 million or more, but less than ¥4 million
- ¥4 million or more, but less than ¥5 million
- ¥5 million or more, but less than ¥6 million
- ¥6 million or more, but less than ¥7 million
- ¥7 million or more, but less than ¥8 million
- ¥8 million or more, but less than ¥9 million
- ¥9 million or more, but less than ¥10 million
- ¥10 million or more, but less than ¥12 million
- ¥12 million or more, but less than ¥15 million
- ¥15 million or more, but less than ¥20 million
- ¥20 million or more

Section 1: Collectivism

Q1 How well does the term "Chinese/Korean" describe you?

- Extremely well
- Somewhat well
- Not too well
- Not at all well

Q2 To what extent do you see yourself as a typical Chinese/Korean person?

- A great deal
- Somewhat
- Not too much
- Not at all

Q3 When talking about Korean people, how often do you say "we" instead of "they"?

- Always
- Sometimes
- Not too often
- Never

Q4 All citizens have the duty to take necessary actions to minimize the spread of the coronavirus.

- Strongly agree
- Somewhat agree
- Somewhat disagree
- Strongly disagree

Section 2: Risk Perception

Q5 I am afraid that I will get the coronavirus.

- Strongly agree
- Somewhat agree
- Somewhat disagree
- Strongly disagree

Q6 I am afraid that people I care about will get the coronavirus.

- Strongly agree
- Somewhat agree
- Somewhat disagree
- Strongly disagree

Section 3: Policies Preference

Q7 Prohibit all non-essential movement of people outside of their homes and ban all large gatherings. Do you support or oppose this policy?

- Strongly support
- Somewhat support
- Somewhat oppose
- Strongly oppose

Q8 Prohibit all people – including Israeli citizens – currently outside the country from re-entering the country.    Do you support or oppose this policy?

- Strongly support
- Somewhat support
- Somewhat oppose
- Strongly oppose

Q9 Prohibit all non-citizens – except legal permanent residents – currently outside the country from entering the country.

Do you support or oppose this policy?

- Strongly support
- Somewhat support
- Somewhat oppose
- Strongly oppose

Q10 Prohibit all people from publicly spreading information about the virus that could have adverse health consequences, including downplaying the virus's danger or inducing panic.

Do you support or oppose this policy?

- Strongly support
- Somewhat support
- Somewhat oppose
- Strongly oppose

Q11 To prohibit Chinese/Korean residents from working or opening businesses.

Do you support or oppose this policy?

- Strongly support
- Somewhat support
- Somewhat oppose
- Strongly oppose

Q12 Suspend all non-essential public or government services, including all primary and secondary schools.

Do you support or oppose this policy?

- Strongly support
- Somewhat support
- Somewhat oppose
- Strongly oppose

Q13 Take over relevant business and seize useful property, including pharmaceutical companies or spaces typically used for government gatherings.

Do you support or oppose this policy?

- Strongly support
- Somewhat support
- Somewhat oppose
- Strongly oppose

Q14 Suspend all religious services or gatherings, including regular church services.

Do you support or oppose this policy?

- Strongly support
- Somewhat support
- Somewhat oppose
- Strongly oppose

Q15 Require some people to work for the government performing healthcare tasks for which there are shortages, like assisting in hospitals or distributing medical supplies.

Do you support or oppose this policy?

- Strongly support
- Somewhat support
- Somewhat oppose
- Strongly oppose

Q16 Detain any individual exhibiting coronavirus-like symptoms and quarantine them in a government facility for at least two weeks.

Do you support or oppose this policy?

- Strongly support
- Somewhat support
- Somewhat oppose
- Strongly oppose

Q17 Permit the government to monitor cellular devices of persons suspected of carrying the Corona virus, and to use their location services to: (1) warn persons with whom they have been in contact; And (2) monitor their movement to ensure they do not violate the isolation instructions.

Do you support or oppose this policy?

- Strongly support
- Somewhat support
- Somewhat oppose
- Strongly oppose

Q18 Instruct the IDF to enforce measures such as banning social gatherings, closing business and isolating them.

Do you support or oppose this policy?

- Strongly support
- Somewhat support
- Somewhat oppose
- Strongly oppose

Section 4: Political tendencies

Q19 Fighting the coronavirus is more important than upholding the law.

- Strongly agree
- Somewhat agree
- Somewhat disagree
- Strongly disagree

Q20 Fighting the coronavirus is more important than upholding the Basic Laws.

- Strongly agree
- Somewhat agree
- Somewhat disagree
- Strongly disagree

Q21 I would feel comfortable giving extraordinary power to the government to allow it to combat the coronavirus.

- Strongly agree
- Somewhat agree
- Somewhat disagree
- Strongly disagree

Q22 Fighting the coronavirus is more important than economic growth.

- Strongly agree
- Somewhat agree
- Somewhat disagree
- Strongly disagree

Q23 Fighting the coronavirus is more important than our civil liberties.

- Strongly agree
- Somewhat agree
- Somewhat disagree
- Strongly disagree

Section 5: Government satisfaction

Q24 Do you approve or disapprove of government’s overall job performance thus far?

- Strongly approve
- Somewhat approve
- Somewhat disapprove
- Strongly disapprove

Q25 Do you approve or disapprove of government 's handling of the coronavirus outbreak?

- Strongly approve
- Somewhat approve
- Somewhat disapprove
- Strongly disapprove
